# Trail Making Test performance in early abstinence from methamphetamine: human evidence for a drug-associated Parkinsonian-like phenotype

**DOI:** 10.3389/fpsyt.2026.1773668

**Published:** 2026-03-23

**Authors:** Alexandru Mihai Dumitrescu, M. Frances Vest, Annette E. Fleckenstein, James C. Patterson, Christina Ledbetter, Nicholas E. Goeders, Jennifer M. Loftis, Elliot Thompson, Katelyn Lofton, Kevin S. Murnane

**Affiliations:** 1 Louisiana Addiction Research Center, Louisiana State University Health Sciences Center at Shreveport, Shreveport, LA, United States; 2 Department of Pharmacology, Toxicology and Neuroscience, Louisiana State University Health Sciences Center at Shreveport, Shreveport, LA, United States; 3 School of Dentistry, University of Utah, Salt Lake City, UT, United States; 4 Interdepartmental Program in Neuroscience, University of Utah, Salt Lake City, UT, United States; 5 Department of Psychiatry and Behavioral Medicine, Louisiana State University Health Sciences Center at Shreveport, Shreveport, LA, United States; 6 Center for Brain Health, Louisiana State University Health Sciences Center at Shreveport, Shreveport, LA, United States; 7 Department of Neurosurgery, Louisiana State University Health Sciences Center at Shreveport, Shreveport, LA, United States; 8 Research and Development Service, Veterans Affairs Portland Health Care System, Portland, OR, United States; 9 Department of Psychiatry, Oregon Health and Science University, Portland, OR, United States; 10 Department of Behavioral Neuroscience, Oregon Health and Science University, Portland, OR, United States

**Keywords:** cognitive flexibility, executive dysfunction, methamphetamine misuse, Parkinsonian-like phenotype, Trail Making Test

## Abstract

**Introduction:**

Methamphetamine misuse is associated with elevated rates of Parkinson’s disease (PD), and both conditions degrade fronto-striatal circuitry, primarily demonstrated in animal and post-mortem human studies. Despite this, few clinical studies have examined overlapping presentation, or whether long-term methamphetamine users exhibit a Parkinsonian-like phenotype. To examine whether recently abstinent individuals with methamphetamine use disorder (MUD) show Parkinsonian-like cognitive inflexibility, and whether these deficits vary by sex or patterns of use.

**Methods:**

Forty-nine individuals with MUD (26 males, 23 females) were recruited from a 30-day residential treatment program and compared with thirty controls (16 males, 14 females). Cognitive flexibility was assessed using the Trail Making Task (TMT), a task sensitive to fronto-striatal deficits in PD. Between-group differences were tested with a two-way between-groups MANCOVA, within-group sex effects with a two-way within-group MANCOVA, and linear regression evaluated the influence of sex and drug intake patterns on PD-like presentation.

**Results:**

Both sexes in the methamphetamine group showed significant TMT deficits relative to controls, consistent with PD populations. Age of first use, duration, and amount of methamphetamine used did not impact performance. Intravenous use, however, was linked to more TMT errors in females but not males.

**Discussion:**

These findings support literature suggesting methamphetamine use resembles aspects of an early Parkinsonian-like phenotype. To our knowledge, this is among the first studies to show PD-like presentation in individuals with MUD, highlighting that women who inject methamphetamine may face disproportionate PD risk. As cognitive inflexibility can hinder treatment engagement, comprehensive interventions for MUD may need to address these deficits.

## 1 Introduction

Methamphetamine is a highly addictive central nervous system psychostimulant, persistently dysregulating the brain, particularly in regions that regulate drug-seeking behavior (1, 2). This dysregulation likely contributes to the difficulty in remaining abstinent from methamphetamine following sustained use (3). In the United States, methamphetamine use has increased significantly, becoming a major contributor to drug-related mortality (4), with over 2.4 million individuals reporting past-year use by 2024 (5). These estimates likely understate prevalence, as national surveys often underrepresent certain populations (6), highlighting the need to better understand methamphetamine’s impact on brain function and behavior.

Beyond its public health burden, methamphetamine misuse has been linked to long-term neurological consequences, most notably an increased risk of Parkinson’s disease (PD). Epidemiological evidence suggests that individuals with methamphetamine use disorder (MUD) have up to a 76% higher risk of developing PD over long-term follow-up, compared to controls and other stimulants like cocaine (7, 8). Meta-analytic and large-scale reviews further confirm this association, reporting more than a three-fold increase in PD risk and highlighting plausible neurobiological mechanisms underlying methamphetamine-induced dopaminergic injury (9, 10). Some studies suggest that women may experience greater risk than men, indicating possible sex-dependent vulnerability (8, 11). While clinical studies directly assessing PD risk in MUD remain limited, converging preclinical evidence demonstrates mechanistic overlap between methamphetamine neurotoxicity and PD pathology (8, 12). Chronic or high-dose exposure produces dopaminergic neurotoxicity, striatal dopamine depletion, and executive impairments in rodents and nonhuman primates (13–16), which parallels early PD before motor symptom onset (17, 18). Moreover, direct administration of methamphetamine induces Parkinsonian-like phenotypes in animal models (14, 19). Together, these data highlight potential convergence between MUD and PD through dopamine-dependent fronto-striatal dysfunction (20–22).

Framing MUD within this “early Parkinsonian-like” model highlights a dimension of methamphetamine-related neurocognitive impairment that has been underappreciated: the convergence with prodromal PD features, where cognitive and subtle motor deficits precede overt motor disability (20–23). Importantly, this work does not suggest methamphetamine use directly causes PD or advanced motor decline. Instead, the goal is to investigate whether chronic methamphetamine exposure induces a PD-like phenotype expressed primarily through deficits in executive function, particularly cognitive flexibility, before motor impairment develops. Rather than establishing clinical equivalence between MUD and PD, the present study seeks to determine whether individuals with MUD exhibit impairment on a cognitive measure known to be sensitive to early PD-related fronto-striatal dysfunction.

Cognitive flexibility, the capacity to adapt behavior in response to changing demands, is critical for adaptive functioning and is often disrupted in substance use disorders, where these impairments reinforce maladaptive patterns and elevate relapse risk (24, 25). Notably, executive dysfunction is also a hallmark feature of PD, with nearly 60% of patients experiencing cognitive impairments within 3–5 years of diagnosis (26–28). Thus, while MUD is often conceptualized as a disorder of motivation, and PD as one of motor decline, emerging work also frames PD as a disorder of motor motivation, in which reduced movement vigor and effort allocation are prominent features, yet both conditions converge mechanistically on fronto-striatal dysfunction and express this through deficits in cognitive flexibility (29–34).

One of the most widely used measures of cognitive flexibility in both clinical and research contexts is the Trail Making Test (TMT). Originally devised as an intelligence task for the United States military (35, 36), the TMT is recognized as a sensitive probe of visual attention, processing speed, and set-shifting, all dependent on fronto-striatal systems (37). It consists of two conditions: TMT-A, which primarily assesses visuospatial search and psychomotor speed, and TMT-B, which adds a set-shifting demand, yielding a derived “shifting cost” score (TMT B–A) that indexes cognitive flexibility (38, 39). Given its strong sensitivity to early Parkinsonian cognitive decline (40–44), the TMT is ideally suited to test whether individuals with MUD show PD-like patterns of impairment.

Demographic and intake-related factors may further shape the emergence of this phenotype. Although methamphetamine was historically associated with younger males and European Americans, recent trends show rising use among females and broader demographer representation, including increases in African American and Native American populations (6, 45–50). In this study, we focused on the impact of sex, age of initiation, duration of use, route of administration, and amount used on TMT performance.

We hypothesized that recently abstinent individuals with MUD (i.e., within one month of cessation) would perform worse than controls in completion time and accuracy across all TMT conditions (TMT-A, TMT-B, and TMT B–A) (32, 41). Further, we expected no sex differences within either group (51). Participants were recruited from two 30−day residential treatment programs and were therefore in early abstinence, as determined by program enrollment and participant self−report. In both programs, patients are under close clinical monitoring and urine drug testing is performed when recent substance use is suspected; however, systematic toxicology data were not available for this study, and precise time since last methamphetamine use was not available because abstinence duration was reported inconsistently, which may introduce additional variability in cognitive performance within this early recovery window. Finally, given the paucity of data on the influence of sex and intake patterns on cognitive outcomes in individuals with MUD, whether active or recently abstinent, we also examined whether performance varied based on key intake metrics: duration of use, route of administration, amount used, and age of first use.

## 2 Materials and methods

### 2.1 Participants

Recruitment occurred in partnership with the Council on Alcoholism and Drug Abuse (CADA) of Northwest Louisiana and the UpRising Addiction Center, two residential addiction treatment centers located in North Louisiana, within the Shreveport-Bossier City metropolitan area. Forty-nine individuals were enrolled in the study, with 23 females and 26 males; age range: 25–45 years, mean age: 35 ± 5 years meeting the inclusion criteria: 1) age 25–55 years old, 2) history of methamphetamine use consistent with the Diagnostic and Statistical Manual of Mental Disorders 5^th^ edition (DSM-5) criteria for a stimulant use disorder, 3) resident of the treatment center for at least 7 days, and 4) English speakers. The exclusion criteria were: 1) meeting criteria for any other substance use disorders with the exception of nicotine and cannabis, 2) unable to sufficiently read and understand the project protocol, 3) unstable medical or psychiatric conditions or disorders – including a clear diagnosis of schizophrenia or Bipolar I disorder (clear from confusion with drug-induced acute states) and history of significant brain injury, stroke, or seizure disorder. Their demographic and clinical characteristics are presented in **Table 1**. To ensure strict adherence to the inclusion/exclusion criteria, individuals with methamphetamine use disorder underwent evaluation using the Quick Structured Clinical Interview from DSM-5 Disorders (QuickSCID-5) by proficient psychiatry residents (52).

**Table 1:** Demographic and clinical characteristics. Values are presented as mean ± SD (standard deviation) or as number and percentage for controls and methamphetamine groups.

Variables	Controls (Ctrl)		Methamphetamine group (Meth)		*p*-value			*Cramér’s V value*		
	F (N = 14)	M (N = 16)	F (N = 23)	M (N = 26)	Ctrl (F *vs.* M)	Meth (F *vs.* M)	Ctrl vs. Meth	Ctrl (F *vs.* M)	Meth (F *vs.* M)	Ctrl vs. Meth
Demographics										
*Age (years)*, *mean (SD)*	35 ± 10	31 ± 5	35 ± 4	35 ± 6	.560	.770	**.040**			
Educational level, N					.403	.392	**<.001**	.353	.334	.814
*Below 8 years*	–	–	3	3						
*9–11 years*	–	–	5	11						
*High school graduate or GED*	1	1	10	11						
*Some college*	7	3	2	1						
*Associate’s degree*	1	2	2	–						
*Bachelor’s degree*	4	9	1	–						
*Master’s degree*	1	1	–	–						
Race, N					.570	.611	.087	.292	.192	.320
*European American*	11	14	22	22						
*Native American*	–	–	1	3						
*African American*	1	2	–	1						
*White hispanic*	1	–	–	–						
*Asian*	1	–	–	–						
Negative emotional states, mean (SD)										
*Depression*	6.85± 8.67	3.56 ± 5.85	10.56 ± 9.71	9.34 ± 7.32	.46	.92	**.008**			
*Anxiety*	4.92 ± 5.69	4.5 ± 4.74	10 ± 8.96	10.38 ± 7.76	.77	1.00	**.003**			
*Stress*	11.14 ± 4.83	7.12 ± 5.18	14 ± 9.89	14.53 ± 8.45	**.05**	1.00	**.008**			
Psychiatric conditions, N (%)										
*Yes*	2 (14.3%)	3 (18.8%)	12 (52.2%)	9 (34.6%)	.75	.21	**.02**			
*No*	12 (85.7%)	3 (81.2%)	11 (47.8%)	17 (65.4%)						
Route of administration, N					–	**.042**	–	–	.329	–
*Inhalation*	–	–	17	11						
*Intravenous*	–	–	6	14						
*Oral ingestion*	–	–	–	1						
*Intranasal*	–	–	–	–						
First methamphetamine use										
*Years*, *mean (SD)*	–	–	21 ± 6	19 ± 7	–	.36	–			
Duration of methamphetamine use										
*Years*, *mean (SD)*	–	–	12 ± 8	16 ± 8	*-*	.08	*-*			
Amount of methamphetamine used										
*Grams/day*, *mean (SD)*	–	–	1.78 ± 1.09	2.97 ± 2.85	–	.07	–			
Co-occurring cannabis use disorder, N (%)										
*Yes*	–	–	6 (26.1%)	10 (38.46%)	–	.36	–			
*No*	–	–	17 (73.9%)	16 (61.54%)	–		–			
Tobacco use, N (%)										
*Yes*	8 (57.14%)	7 (43.75%)	17 (73.91%)	22 (84.62%)	.47	.36	.**006**			
*No*	6 (42.86%)	9 (56.25%)	6 (26.09%)	4 (15.38%)						

Between-group and within-group sex differences are highlighted in bold. For categorical variables, chi-square or Fisher’s exact tests were used as appropriate. *P*-values for depression, anxiety, and stress subscales were adjusted using the Holm–Bonferroni procedure. *F, females; M, males; Ctrl, controls; Meth, methamphetamine group; N, number.*

Thirty controls were recruited from the local community between June 2021 and March 2023, with 14 females and 16 males; age range: 25–54 years, mean age: 33 years ± 8 meeting the inclusion criteria: 1) age 25–55 years old, 2) English speaker. Exclusion criteria included: 1) meeting DSM-5 criteria for any substance use disorder (except nicotine and cannabis); 2) unstable medical or psychiatric conditions or disorders, including schizophrenia or Bipolar I disorder; 3) history of significant brain injury, stroke, or seizure disorder; and 4) inability to understand the informed consent, study purpose, or procedures.

Psychiatric conditions were defined as prior clinician-diagnosed DSM-5 psychiatric disorders reported by participants. All participants were asked whether they had previously received a formal psychiatric diagnosis. In the methamphetamine group, diagnoses were verified using the QuickSCID-5 to distinguish primary psychiatric disorders from substance-induced acute states.

This study was approved by the Louisiana State University Health Science Center - Shreveport Institutional Review Board. All participants provided written, informed consent in accordance with the Declaration of Helsinki.

An *a priori* power analysis was conducted using G*Power (version 3.1.9.7) (53), which determined that a minimum of 22 participants per group would be required to detect a large effect size (f = 0.40) with 80% power at an alpha level of 0.05.

### 2.2 Clinical assessments

#### 2.2.1 Drug use history

To capture the nature of participants’ methamphetamine use, they were asked a series of questions regarding their drug use history. These questions included primary route of administration, age of first use, duration of use, amount used per day, and duration of abstinence. Although duration of abstinence was queried, responses were incomplete and inconsistently reported, so precise time since last methamphetamine use could not be reliably quantified and was not included in the primary analyses. In one of the residential programs, some participants also had admission toxicology results that provided approximate indicators of recent methamphetamine exposure; however, these measures were not collected in a standardized manned and were therefore not used as analytic variables.

These methamphetamine-use variables, including amount used per day, were obtained during clinical screening and reflect participants’ typical daily consumption during their period of regular methamphetamine use, rather than use within a specific fixed window (e.g., past 30 days or past year).

Tobacco use was recorded during clinical screening as part of the substance use history (yes/no regular use) and was not collected as a standardized quantitative measure anchored to a defined reporting period.

#### 2.2.2 Depression Anxiety Stress Scales (DASS-42)

The DASS-42 assesses negative emotional states across three domains: depression, anxiety, and stress, with 14 items per subscale (54, 55). Each of the 42 questions is scored on a 4-point scale ranging from 0 (“Did not apply to me at all”) to 3 (“Applied to me very much, or most of the time”). Scores for depression, anxiety and stress are calculated by summing the scores for the relevant items. This self-report questionnaire was administered individually on a laptop in a quiet, distraction-free room.

### 2.3 Experimental procedure

The TMT was administered using a laptop computer with a connected mouse, using the Inquisit 5.0 software (56) in a quiet room free of distractions. An initial demonstration was performed with a given sample trial before each TMT condition (TMT-A and TMT-B) to familiarize the participants and confirm they understood the instructions. In TMT-A, participants were instructed to use the mouse to connect 25 randomly scattered nodes numbered 1 to 25 in ascending order (i.e., 1-2-3…), whereas in TMT-B, they had to connect alternating nodes of numbers and letters in ascending order (i.e., 1-A-2-B-3-C…). Participants were asked to complete each TMT condition as quickly as possible without compromising the accuracy of their performance. Both total completion time (seconds) and number of errors (i.e., going responding in the incorrect order) made during each TMT condition were recorded.

### 2.4 Statistical analysis

All analyses were carried out using IBM^®^ SPSS^®^ Statistics software (version 29.0.2.0. IBM Corporation; Armonk, NY). The level of statistical significance was set at α = .05 (two-sided).

Categorical variables were analyzed using chi-square tests. For comparisons involving educational level, race, and route of methamphetamine administration, Fisher’s exact tests were used due to sparse cell counts and violation of chi-square assumptions (i.e., expected frequencies < 5 in more than 20% of cells or expected counts < 1). Psychiatric conditions, tobacco use, and co-occurring cannabis use disorder were analyzed using chi-square tests. Cramér’s V was calculated as a measure of effect size for categorical comparisons.

Negative emotional states were assessed using the depression, anxiety, and stress subscales. To control for multiple comparisons across these three related subscales within each family of tests, *p-*values were adjusted using the Holm–Bonferroni method (family-wise *α* = .05). Holm corrections were applied separately for between-group comparisons and within-group sex comparisons.

When significant multivariate effects were observed in the TMT analysis, follow-up pairwise comparisons of estimated marginal means were performed with Bonferroni adjustment to control for multiple comparisons.

#### 2.4.1 Between-group comparisons

Independent-samples t-tests (two-sided) were used to examine differences in age and in depression, anxiety, and stress scores between the methamphetamine group and controls. Group differences in educational level and race were assessed using Fisher’s exact tests due to sparse cell counts. Differences in psychiatric conditions, and tobacco use were evaluated using chi-square tests. Cramér’s V was calculated as a measure of effect size for categorical comparisons.

A two-way multivariate analysis of covariance (MANCOVA) (57) was performed to investigate the effects of group (methamphetamine and controls) and TMT condition (TMT-A, TMT-B, and TMT B-A) on total completion time (seconds) and number of errors as the dependent variables. Covariates included age, educational level, psychiatric conditions, depression, anxiety, stress scores, and tobacco use. Pairwise comparisons of estimated marginal means were adjusted using Bonferroni correction.

Preliminary assumption testing was carried out to check for normality, linearity, univariate and multivariate outliers, homogeneity of variance covariance matrices, and multicollinearity, with no violations noted. Effect sizes were calculated using partial eta square (*η_p_^2^*) as proposed by (58), representing the variance in the dependent variable explained by the independent variable.

#### 2.4.2 Within-group sex comparisons

Within each group, sex differences in age and in depression, anxiety, and stress scores were examined using independent-samples t-tests (two-sided), with Holm-adjusted *p-*values reported for the three negative emotional state subscales. Sex differences in educational level and race were assessed using Fisher’s exact tests due to sparse cell counts. Differences in psychiatric conditions and tobacco use were evaluated using chi-square tests. Cramér’s V was calculated as a measure of effect size for categorical comparisons. In the methamphetamine group only, sex differences in co-occurring cannabis use disorder were assessed using chi-square tests. Additional comparisons in the methamphetamine group included sex differences in methamphetamine use variables (age of first use, duration of use, and amount used in grams/day) using independent-samples t-tests, and differences in route of methamphetamine administration using Fisher’s exact test.

A two-way within-group MANCOVA was conducted separately in both the methamphetamine group and the control group to examine differences by sex and TMT condition on total completion time (seconds) and number of errors. Preliminary assumption testing indicated no violations, and partial eta squared (*η_p_^2^*) was used to assess effect sizes. To account for individual differences, the route of methamphetamine administration was included as a covariate in the methamphetamine group, while stress was used as a covariate in the control group. In addition, exploratory comparisons of self-reported daily methamphetamine use (grams/day) were conducted within the methamphetamine group to provide contextual information for interpreting cognitive outcomes; these included comparisons (a) between males and females within each route of administration (inhalation and intravenous) and (b) between inhalation and intravenous use within each sex. These analyses were not prespecified and should be considered exploratory. *Post hoc* comparisons of estimated marginal means were adjusted using Bonferroni correction to control for family-wise error.

#### 2.4.3 Stepwise multiple linear regression analysis

Stepwise multiple linear regression was performed to identify methamphetamine-related variables predictive of performance on each TMT condition. Based on previous findings (59, 60), the selected predictors included age of first use, duration of use, daily amount used, and route of administration. Given the type-I error in stepwise procedures (61, 62), the number of predictors was intentionally limited to four, with caution applied in interpreting results.

## 3 Results

### 3.1 Between-group comparisons

#### 3.1.1 Demographic and clinical data

**Table 1** presents demographic and clinical characteristics for controls and methamphetamine users. Compared to controls, the methamphetamine group had significantly lower educational status, a higher prevalence of psychiatric conditions, and elevated depression, anxiety, and stress scores, and greater tobacco use. They were also older than the control group.

#### 3.1.2 TMT performance

Assumptions for homogeneity of variance-covariance matrices were met, as indicated by a non-significant Box’s M test (Box’s M = 95.265, *p* = .156). Similarly, Levene’s test confirmed homogeneity of variance for both dependent variables—total completion time and number of errors (*p* >.05).

After adjusting for age, education, psychiatric conditions, tobacco use, depression, anxiety, and stress scores, a significant interaction was found between group (methamphetamine group vs. control group) and TMT condition (TMT-A, TMT-B, and TMT B-A) on the combined dependent variables, Fisher’s *F (4*, 446) = 3.666, *p* = .006; Wilks’s Lambda λ = .937. Despite a small effect size (*η_p_^2^* = .032), both main effects were significant: group, *F (2*, 223) = 23.772, *p* <.0001, and TMT condition, *F (4*, 446) = 81.141, *p* <.0001. Notably, none of the covariates were significantly associated with the combined dependent variables.

Follow-up univariate analyses (ANOVA) revealed significant group differences in total completion time, *F (1*, 224) = 41.988, *p* <.0001, *η_p_^2^* = .158, and number of errors, *F (1*, 224) = 16.058, *p* <.0001, *η_p_^2^* = .067. The methamphetamine group took significantly longer to complete the TMT and made more errors than controls. The effect size for completion time (*η_p_^2^* = .158) suggests it was a stronger distinguishing factor between groups than error rate (*η_p_^2^* = .067).

TMT condition also significantly influenced total completion time, *F (2*, 224) = 202.666, *p* <.0001, *η_p_^2^* = .644, and number of errors, *F (2*, 224) = 8.917, *p* <.001, *η_p_^2^* = .074. Pairwise comparisons showed that TMT-B had the longest completion time (*p* <.0001) and highest error rate (*p* <.001), followed by TMT-A, which took significantly longer than TMT B-A (*p* <.0001) but did not differ in errors (*p* = .98). Again, total completion time (*η_p_^2^* = .644) had a stronger impact than error count.

**Figure 1:**
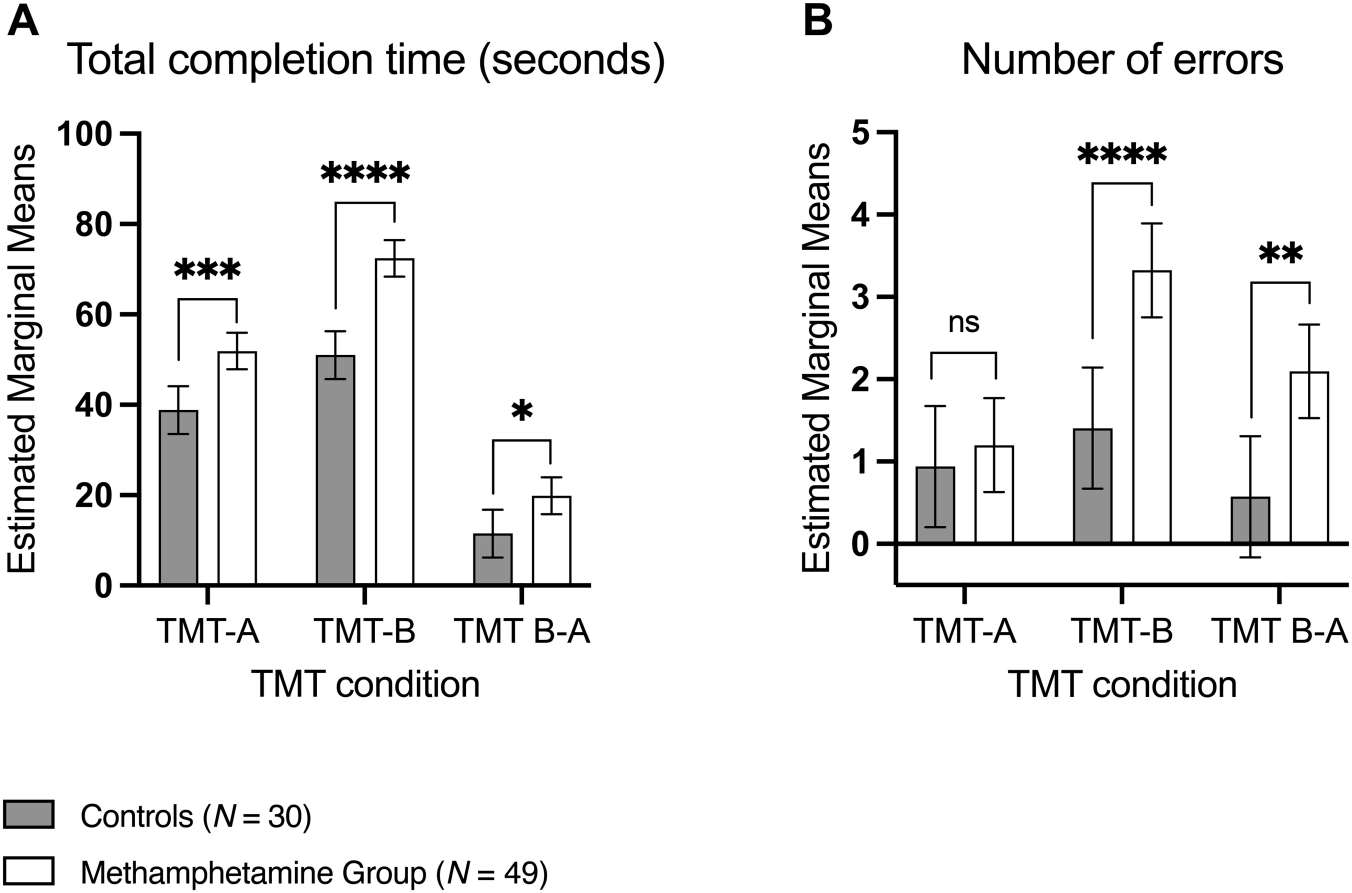
Adjusted marginal means and 95% confidence intervals for **(A)** total completion time (seconds) and **(B)** number of errors; distribution by group and condition. Significance levels reflect Bonferroni-adjusted pairwise comparisons of estimated marginal means. *TMT, Trail Making Test.*^ns^*p* >.05, *p <.05, **p <.01, ***p <.001, ****p <.0001.

A significant interaction between group and TMT condition was observed for both total completion time, *F (2*, 224) = 4.048, *p* = .019, *η_p_^2^* = .035, and number of errors, *F (2*, 224) = 3.571, *p* = .030, *η_p_^2^* = .031. The methamphetamine group consistently took longer across all TMT conditions (*p* <.0001). They also committed significantly more errors in TMT-B and TMT B-A (*p* <.0001), but not in TMT-A (*p* = .591) (**Table 2**, **Figure 1**).

**Table 2:** Adjusted marginal means (and standard errors) of the total completion time and number of errors by group and condition, after controlling for the effect of age, educational level, psychiatric conditions, tobacco use, depression, anxiety and stress scores.

		Controls (*N* = 30)			Methamphetamine group (*N* = 49)			Controls vs. Methamphetamine group (*p*-value)		
Neurocognitive performance (Dependent variable)		TMT A	TMT B	TMT B-A	TMT A	TMT B	TMT B-A	TMT A	TMT B	TMT B-A
Total completion time (seconds)	Mean (SE)	38.845 (2.675)	51.018 (2.675)	11.503 (2.675)	51.906 (2.065)	72.425 (2.065)	19.891 (2.065)	**<.001**	<**.0001**	**.016**
	95% CI (Lower Bound)	33.574	45.747	6.231	47.837	68.355	15.821			
	95% CI (*Higher Bound*)	44.117	56.290	16.774	55.976	76.494	23.960			
Number of errors	Mean (SE)	.940 (.374)	1.406 (.374)	0.573 (.374)	1.200 (.289)	3.323 (.289)	2.098 (.289)	.591	<**.0001**	**<.002**
	95% CI (Lower Bound)	.203	.670	-.164	.632	2.754	1.530			
	95% CI (*Higher Bound*)	1.676	2.143	1.309	1.769	3.891	2.667			

Between-group differences are highlighted in bold. *P*-values reflect Bonferroni-adjusted pairwise comparisons of estimated means. *TMT, Trail Making Test; SE, standard error; CI, confidence interval.*

### 3.2 Within-group sex comparisons

#### 3.2.1 Demographic and clinical data

Demographic and clinical variables for females and males in both groups are detailed in **Table 1**. Among the methamphetamine group, females primarily used inhalation, while males predominantly used intravenous injection. In the control group, females reported significantly higher stress scores than males. Although not part of the primary analysis, exploratory comparisons of self-reported daily methamphetamine use (grams/day) within the methamphetamine group indicated no meaningful dose differences by sex or route: for inhalation, females used 1.68 ± 1.17 grams/day and males 2.87 ± 2.86 grams/day, and for intravenous use, females used 2.08 ± 1.07 grams/day and males 2.89 ± 2.88 grams/day (all *p* >.05); within each sex, daily amounts were also similar across inhalation and intravenous routes.

#### 3.2.2 TMT performance

In the methamphetamine group, assumptions for homogeneity of variance-covariance matrices were met (Box’s M = 49.182, *p* = .318), and Levene’s test confirmed comparable variance across sexes (*p* >.05).

After controlling for the route of administration, no significant interaction between sex and TMT condition was found for the combined dependent variables, *F (4*, 278) = .240, *p* = .915; Wilks’s Lambda λ = .993 (*η_p_^2^* = .003). However, a significant main effect of TMT condition was observed, *F (4*, 278) = 55.852, *p* <.0001. The main effect of sex showed a trend toward significance, *F (2*, 139) = 2.939, *p* = .056. The route of administration was not significantly associated with the combined dependent variables, *F (2*, 139) = 1.315, *p* = .272.

Follow-up univariate analyses confirmed a significant effect of TMT condition on total completion time, *F (2*, 140) = 127.618, *p* <.0001, *η_p_^2^* = .646, and number of errors, *F*(2, 140) = 11.412, *p* <.0001, *η_p_^2^* = .140. TMT-B had significantly longer completion times than TMT-A and TMT B-A (p <.0001), and TMT-A was longer than TMT B-A (*p* <.0001). For errors, TMT-B had significantly higher means than both TMT B-A (p = .024) and TMT-A (*p* <.0001), while TMT-A and TMT B-A did not differ (*p* = .119).

Sex differences were significant for total completion time, *F*(1, 140) = 5.908, *p* = .016, *η_p_^2^* = .040, with males taking longer than females. No significant difference was found for errors, *F*(1, 140) = .568, *p* = .452, *η_p_^2^* = .004. The only significant sex difference emerged for TMT-B completion time (*p* = .047), whereas no sex differences were observed for errors (all *p* >.05).

In the control group, assumptions of homogeneity were met (Box’s M = 26.668, *p* = .051), and variance was comparable across sexes (*p* >.05). After controlling for stress scores, no significant interaction between sex and TMT condition was found, *F*(4, 164) = .186, *p* = .945; Wilks’s Lambda λ = .991, *η_p_^2^* = .005. A significant main effect of TMT condition was observed, *F*(4, 164) = 33.833, *p* <.0001, while the main effect of sex was not significant, *F*(2, 82) = .147, *p* = .863. Stress scores were also unrelated to TMT performance.

Follow-up analyses confirmed a significant TMT condition effect for total completion time, *F*(2, 83) = 95.121, *p* <.0001, *η_p_^2^* = .696, and number of errors, *F*(2, 83) = 3.394, *p* = .038, *η_p_^2^* = .076. TMT-B took significantly longer than both TMT-A and TMT B-A (p <.0001), and TMT-A was longer than TMT B-A (*p* <.0001). For errors, TMT-B had significantly higher values than TMT B-A (*p* = .033) but did not differ from TMT-A (*p* = .468). Again, total completion time was the primary driver of TMT condition effects.

### 3.3 Stepwise multiple linear regression results

To explore methamphetamine use characteristics as predictors of TMT performance, a stepwise multiple linear regression was conducted with age of first use, duration of use, amount used (grams/day), and route of administration. At the group level, none of these variables significantly predicted completion time or errors across TMT conditions.

**Figure 2:**
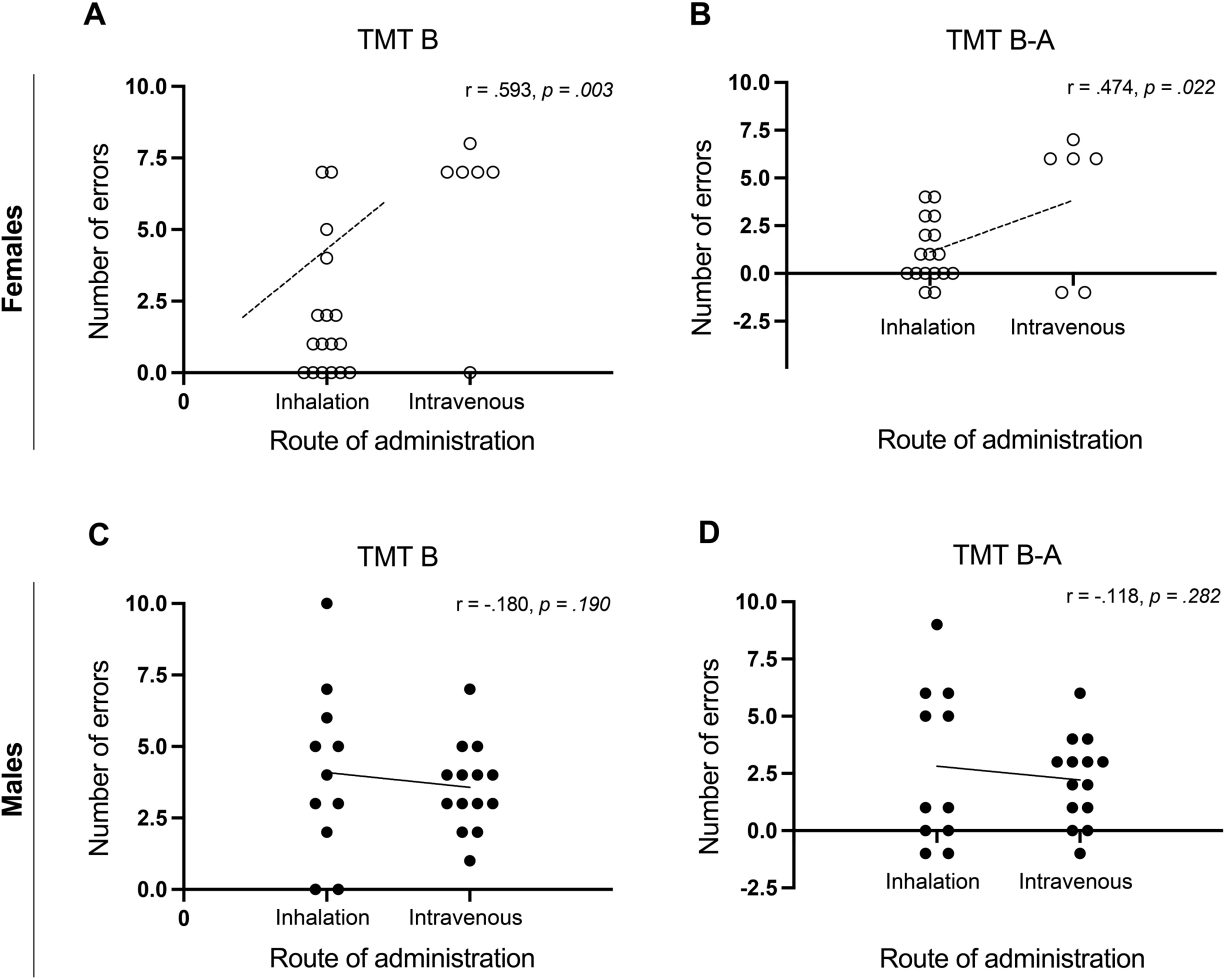
**(A, B)** show the relationship between the number of errors in TMT-B and B-A and the route of methamphetamine use in females, respectively, while **(C, D)** show the same relationships in males.

However, within-group, route of administration significantly predicted error rates in TMT-B for females, *F*(1, 21) = 11.370, *p* = .003, *β* = 0.593, explaining 35.1% of variance (ΔR² = .351) (**Figure 2A**). Intravenous administration was linked to higher errors in TMT-B. A similar effect was found in TMT B-A, *F*(1, 21) = 6.100, *p* = .022, *β* = 0.474, explaining 22.5% of variance (ΔR² = .225) (**Figure 2B**). No such association was found in males (*p* >.05) (**Figures 2C, D**).

## 4 Discussion

Cognitive flexibility, the capacity to adapt to changing demands by switching task sets or strategies, plays a critical role in substance use disorders, influencing maladaptive decision-making and relapse risk (24, 25). Importantly, deficits in this domain are also well-documented in PD, where disruption of dopamine-dependent fronto-striatal circuits compromises executive control (13, 32, 33, 41). Given the shared disruption of dopamine-rich circuits in both PD and MUD, supported by epidemiological evidence of elevated PD risk (7) and preclinical findings of methamphetamine-induced dopaminergic toxicity (13), cognitive flexibility provides a promising cross-condition target for understanding executive dysfunction. Considering this overlap, the present study assessed cognitive flexibility in individuals recently abstinent from methamphetamine compared to controls using the TMT to determine whether individuals with MUD exhibit patterns of inflexibility that resemble what is typically observed in early PD on this task, relative to healthy controls rather than to a PD comparison group.

In line with our hypotheses, the individuals recently abstinent from methamphetamine showed significantly poorer performance on the TMT relative to controls. Because precise time since last methamphetamine use was not available, we could not determine how TMT performance varied across the early abstinence period, and this may have contributed to the within-group variability observed in the methamphetamine sample. Specifically, they exhibited slower total completion times and a higher number of errors in TMT-B and TMT B-A conditions that demand greater cognitive flexibility. These findings align with prior studies showing that the methamphetamine group was significantly slower than the controls in TMT-B (63), suggesting that methamphetamine use is associated with deficits in executive function, particularly cognitive flexibility. In addition, although it is generally accepted that TMT B-A offers a finer index of cognitive flexibility, as it calculates the *shifting cost* (64), to the best of our knowledge, none of the previous work (51, 63, 65–71) has investigated this TMT condition in a methamphetamine group, whether active or recently abstinent, relative to controls. We sought to address this gap by providing the first evidence that individuals meeting criteria for MUD exhibit cognitive inflexibility, as reflected in greater shifting costs, a pattern that parallels those observed in PD (41, 72–77). We suggest that these results reflect impaired cognitive flexibility in the methamphetamine group, which may be an important factor contributing to an individual’s difficulty in initiating and maintaining abstinence and, therefore, may promote continued drug use (78). Furthermore, our findings converge not only with previous studies of human methamphetamine use indicating impairment on other tasks probing cognitive flexibility such as the Wisconsin Card Sorting Task (79, 80), and reversal learning tasks (81, 82), but also with previous preclinical animal studies of other drugs of misuse. In particular, non-human primates that were passively exposed to cocaine exhibited deficits in cognitive flexibility tasks (i.e., reversal learning) when tested later in the absence of drug (83, 84). Similarly, rats with prior experience of cocaine self-administration or passive injections of cocaine, were unusually slow in their cognitive flexibility tasks (85, 86). Additional studies showed that low cognitive flexibility (set-shifting) was associated with increased alcohol consumption in both rodent and non-human primates (87, 88).

Despite increasing evidence of the negative effects of different substances of misuse on the brain, neuropsychological research on cognitive flexibility using TMT in methamphetamine misuse remains sparse, and findings have been inconsistent. Prior studies reporting null findings (51, 67, 68, 71, 89, 90) may have failed to detect group differences due to lower levels of methamphetamine use or shorter use histories relative to the current cohort. However, reporting of methamphetamine use characteristics varied substantially across these and other studies. While some studies clearly quantified abstinence duration, years of use or daily consumption were inconsistently reported. Therefore, the calculated averages for daily consumption and duration of use were derived from the subset of studies that provided sufficient quantitative data for each respective variable. Our methamphetamine group reported substantially higher daily consumption (2.72 ± 2.81 grams/day) compared to the mean levels derived from studies reporting daily use (mean across cited studies: ~0.99 ± 0.65 grams/day) (66, 68, 69, 91, 92). Similarly, our participants had longer histories of use (14 ± 8 years) relative to the mean duration reported in studies providing this information (mean across cited studies: ~6.15 ± 3.46 years) (65, 66, 68, 90, 91). Such higher and prolonged exposure is known to exacerbate neurotoxicity and neuroinflammation (93–96), which may help explain the more pronounced cognitive deficits observed in our sample. Another possibility relates to differences in age of onset: participants in previous studies typically initiated use at an older age (mean across cited studies: 24.60 ± 3.58 years) (68, 91) compared to those in our study (20 ± 7 years). Earlier initiation has been associated with greater vulnerability to executive dysfunction, aligning with evidence that cognitive deficits, particularly in executive control, are common among individuals who misuse methamphetamine (2). Taken together, the higher daily consumption, longer histories of use, and earlier onset of methamphetamine use observed in our sample may help to explain the more pronounced impairments in cognitive flexibility, which appear to persist into the early abstinence phase and, in turn, may increase vulnerability to relapse before sustained recovery can be achieved.

An additional aim of this study was to explore sex-specific effects on cognitive flexibility within the methamphetamine group. While a previous study (51) reported no significant sex differences in cognitive performance among individuals with MUD and their controls, the present findings suggest otherwise. Specifically, males exhibited slower TMT-B completion times compared to females, but no significant sex differences were observed in the number of errors across TMT conditions. This pattern suggests that processing speed deficits may be more pronounced in males, whereas accuracy-related impairments may be more relevant in females, particularly when considering route of administration.

Indeed, the results highlight a sex-specific effect of intravenous methamphetamine use on executive dysfunction. Among females with MUD, intravenous administration was significantly associated with a greater number of errors on both TMT-B and TMT B-A, whereas this pattern was absent in males. To evaluate whether these sex-specific associations could be explained by differences in methamphetamine exposure, exploratory analyses examined self-reported daily methamphetamine use (grams/day) by sex and route. Within each route, females and males reported comparable daily amounts for both inhalation and intravenous use, and within each sex, daily amounts were similar across inhalation and intravenous routes (all *p* >.05), indicating that simple dose differences by sex or route are unlikely to fully account for the observed female-specific association between intravenous use and increased TMT errors. Consistent with this, females showed overall lower use quantities than males at the group level (trend *p* <.07), yet the intravenous route remained specifically linked to greater shifting-related executive dysfunction only in females (97), suggesting a heightened vulnerability to the neurocognitive consequences of more direct administration rather than a mere dose effect.

These findings emphasize the need to consider sex differences in the cognitive consequences of methamphetamine use and highlight how route of administration may differentially affect cognitive flexibility in females. They also highlight the importance of future work to clarify the neurobiological mechanisms that might render females particularly susceptible to the cognitive impact of intravenous methamphetamine, including potential interactions with hormonal, neurovascular, or inflammatory pathways, and to develop tailored interventions aimed at mitigating these impairments (98).

At the neural level, the observed impairment in TMT performance in individuals with MUD may reflect dysfunction in fronto-striatal circuits, particularly the dorsolateral prefrontal cortex and its projections to the basal ganglia. These same circuits are critically disrupted in PD, a condition that is also associated with deficits in executive functioning and cognitive flexibility, particularly in its early stages before motor symptoms are observable (14, 99–104). Notably, neuroimaging work in MUD indicates greater-than-normal age-related cortical gray matter loss and other structural indices of premature brain pathology, consistent with accelerated brain aging in this population. Such age-advanced structural changes prominently involve frontal and striatal regions that support executive control and set-shifting, providing a further point of convergence between MUD and age-related neurodegenerative conditions such as PD (105, 106). In both disorders, dopaminergic dysregulation is a central feature, which may partly account for the overlapping TMT performance profiles.

However, dopaminergic mechanisms alone are unlikely to fully account for the cognitive impairments associated with MUD. Growing evidence indicates that methamphetamine also disrupts glutamatergic and GABAergic neurotransmission (107–111). These neurotransmitter systems play key roles in modulating prefrontal and striatal function and are essential for flexible, goal-directed behavior. Preclinical studies further support this view, demonstrating that psychostimulant-induced alterations in glutamate and GABA pathways impair cognitive flexibility and executive processes (112–114). Altogether, the evidence suggests that executive dysfunction in MUD results from a complex interplay of dopaminergic, glutamatergic, and GABAergic alterations within fronto-striatal networks. Future research should clarify the relative contributions of these systems to the neurobiological mechanisms underlying cognitive deficits in this population.

Finally, the clinical implications of impaired cognitive flexibility in MUD extend beyond cognitive deficits per se, particularly in relation to treatment outcomes. Cognitive behavioral therapies (CBT), one of the primary treatment interventions for substance use disorders, require patients to monitor and adapt maladaptive thought and behavior patterns. This process is cognitively demanding and can be especially challenging for individuals with executive dysfunction, a difficulty also seen in PD (32, 40, 78, 115). These parallels suggest that cognitive inflexibility may limit treatment responsiveness in MUD just as it does in PD, reinforcing the need to identify and target these impairments directly. Specifically, deficits in this domain may hinder an individual’s responsiveness to CBT, potentially affecting treatment outcomes and increasing relapse risk (116). Interventions aimed at enhancing cognitive flexibility, whether through targeted cognitive rehabilitation or adjunctive approaches, may therefore improve the efficacy of CBT among this population. Moreover, emerging strategies such as exercise-based programs or melatonin supplementation show preliminary promise in restoring cognitive flexibility and could provide novel avenues for supporting cognitive recovery in methamphetamine users (117, 118).

## 5 Limitations

This study has several limitations. First, the methamphetamine group had lower educational levels and higher depression, anxiety, and stress scores than controls, consistent with previous findings that individuals with MUD tend to have lower education and more psychiatric comorbidities (51, 65, 66, 68, 119, 120). Preclinical studies suggest that methamphetamine disrupts brain regions involved in stress regulation, such as the amygdala, hippocampus, and hypothalamus (121, 122), which may contribute to these elevated symptoms. To minimize confounding, we statistically controlled for age, education, and psychiatric symptoms when assessing cognitive flexibility. Second, all individuals with MUD were recruited from two 30−day residential programs and were in early abstinence based on participant self−report within that setting. Although patients are closely monitored in these programs and are drug tested when recent use is suspected, systematic toxicology verification was not available for this study; precise time since last methamphetamine use was not available, so we could not determine how TMT performance varied across the early abstinence period, and this may have contributed to the within−group variability observed in the methamphetamine sample. Future work should recruit individuals with longer periods of abstinence, including those living in the community rather than residential settings, to characterize more stable cognitive phenotypes over the course of recovery. Third, age is a major driver of PD progression, and our use of relatively broad age ranges may have limited our ability to finely model age−related effects; larger samples will be needed to stratify participants into narrower (e.g., 10−year) age bands to better disentangle age, disease duration, and methamphetamine−related influences on PD−related features. Forth, cannabis use disorder was present only in the methamphetamine group and absent in controls, meaning it could not be modeled as an independent covariate in between−group comparisons and instead reflects part of the broader clinical profile of the methamphetamine group. Finally, we did not assess genetic risk factors or environmental exposures (e.g., pesticides, heavy metals, industrial solvents) that are known to influence PD risk and progression, so these unmeasured contributors may also have affected the observed PD−related phenotype; integrating genetic and environmental risk markers in future studies would help clarify their role.

## 6 Conclusion

Altogether, these findings not only deepen our understanding of cognitive impairments in MUD but also support a broader neurobiological framework in which fronto-striatal disruptions, common to both MUD and PD, may underlie critical treatment-relevant deficits. Our findings also highlight the need for sex-specific and pattern-of-use-specific interventions, which could improve treatment outcomes. Given that cognitive inflexibility may limit responsiveness to cognitively demanding treatments such as CBT, integrating adjunctive approaches that enhance cognitive flexibility, particularly for women who use methamphetamine via intravenous administration, could help maximize the therapeutic benefits of CBT. More broadly, this work reinforces the importance of targeting cognitive impairments as a critical strategy for reducing relapse risk and promoting sustained abstinence in MUD.

## Data Availability

The raw data supporting the conclusions of this article are not publicly available due to ethical and legal restrictions related to participant privacy and institutional policy. De-identified data may be made available to qualified researchers upon reasonable request, contingent on approval from the study’s principal investigator and compliance with LSU Health Shreveport policies. This includes the potential establishment of a Collaborative Research Agreement or Material Transfer Agreement. Requests should be directed to Dr. Kevin Murnane (kevin.murnane@lsuhs.edu).
